# Changes of blood gas and serum indexes in patients with acute heart failure complicated with respiratory failure treated by noninvasive ventilator

**DOI:** 10.12669/pjms.38.7.6526

**Published:** 2022

**Authors:** Yuan Wu, Xin Hong, Ni Yang, Ru Zhang, Yu Shi

**Affiliations:** 1 Yuan Wu, Department of Emergency, the Second Affiliated Hospital of Xi’an Jiaotong University, 157, Xiwu road, Xi’an 710004, Shaanxi Province, P.R. China; 2 Xin Hong, Department of Emergency, the Second Affiliated Hospital of Xi’an Jiaotong University, 157, Xiwu road, Xi’an 710004, Shaanxi Province, P.R. China; 3 Ni Yang, Department of Emergency, the Second Affiliated Hospital of Xi’an Jiaotong University, 157, Xiwu road, Xi’an 710004, Shaanxi Province, P.R. China; 4 Ru Zhang, Department of Emergency, the Second Affiliated Hospital of Xi’an Jiaotong University, 157, Xiwu road, Xi’an 710004, Shaanxi Province, P.R. China; 5 Yu Shi, Department of Emergency, the Second Affiliated Hospital of Xi’an Jiaotong University, 157, Xiwu road, Xi’an 710004, Shaanxi Province, P.R. China

**Keywords:** Noninvasive ventilator, Auxiliary ventilation, Acute heart failure, Respiratory failure, Blood gas index, Serum index

## Abstract

**Objective:**

To observe the changes of blood gas and serum indexes in patients with acute heart failure complicated with respiratory failure treated by noninvasive ventilator-assisted ventilation.

**Methods:**

The records of patients with acute heart failure and respiratory failure, treated in our hospital from June 2019 to June 2021, were selected and stratified based on the received therapy. Of a total of 168 patients, 81 received continuous low flow oxygen therapy (control group) and 87 received noninvasive ventilator (NIV)-assisted ventilation therapy (NIV group). The blood gas and serum indexes of the two groups were compared and analyzed.

**Results:**

After 48 hours of treatment, the blood oxygen saturation (SaO_2_), arterial partial pressure of oxygen (PaO_2_) and oxygenation index (PaO_2_/FiO_2_) in patients in the NIV group were higher than those in the control group, while the expression of arterial partial pressure of carbon dioxide (PaCO_2_) was lower than that in the control group (*P*<0.05). After 24 hours and 48 hours of treatment, there was an increase in the expression of N-terminal pro-B-type natriuretic peptide (NT-pro BNP) and Cardiac troponin I (cTnI) in both groups. The expression of NT-pro BNP and cTnI in the NIV group was significantly lower than that in the control group (*P*<0.05).

**Conclusion:**

In patients with acute heart failure and respiratory failure, noninvasive ventilator-assisted ventilation results in improved blood gas analysis indexes and lower levels of heart disease markers, NT-pro BNP and cTnI.

## INTRODUCTION

Heart failure is one of the common clinical diseases, it is predominantly caused by the pathological changes in the cardiac function or structure and is mainly characterized by the sudden decrease of cardiac output, resulting in the insufficient perfusion of tissues and organs.^1^ Heart failure has a rapid onset, and is manifested by gray complexion, severe dyspnea and forced sitting. It is often accompanied by hypoxemia, resulting in brain and organ failure, which severely affects patient’s health and quality of life.^2^

After the occurrence of acute heart failure, the ejection fraction of the heart and the systolic function are significantly reduced, resulting in congestion of the pulmonary circulation and respiratory failure.^3^ Recent research shows that in the treatment of patients with acute heart and respiratory failure, the focus is to timely correct the symptoms of hypoxia, effectively improve the blood oxygen saturation and enhance the lung compliance, to save patient’s life and improve the prognosis.^4^ At this stage, the clinical treatment of acute heart failure combined with respiratory failure is mainly continuous low flow oxygen inhalation.

Although it can alleviate the symptoms to a certain extent, the effect needs to be improved.^5^ Recent advances in the methods of noninvasive ventilation (NIV), made noninvasive ventilator-assisted ventilation technology a method of choice in treating patients with heart and respiratory failure as a convenient, non-invasive and safe technique. It can help to reduce rates of endotracheal intubation, shortens ventilation time, promote the work of patients’ cardiomyocyte tissue, to quickly improve energy metabolism, alleviate the degree of respiratory failure and to improve the overall success rate of the treatment.^6^ In recent years, our hospital has applied noninvasive ventilator-assisted ventilation to the treatment of patients with acute heart failure complicated with respiratory failure. The main goal of this study was to retrospectively analyze and evaluate the application effect and clinical value of noninvasive ventilator-assisted ventilation using blood gas analysis index and serum index.

## METHODS

The records of 168 patients (90 males and 78 females) with acute heart failure and respiratory failure that were treated in our hospital from June 2019 to June 2021 were retrospectively selected. The medical records showed that all patients received symptomatic treatment such as bronchodilators, cardiotonics and diuretics.

### Inclusion criteria:

- Meets the relevant diagnostic criteria of “acute heart failure” and “respiratory failure”;^7^-^8^
- Age range of 18 ≤ 80 years;
- NYHA classification of cardiac function of Grade- IV;
- Indications for noninvasive ventilator-assisted ventilation;
- Complete medical records.

### Exclusion criteria:

- Severe thoracic trauma or arrhythmia;
- Endotracheal intubation due to illness;
- Secretions in the airway, and risk of asphyxia;
- Severe organ dysfunction;
- Mental, directional or cognitive impairment;
- Malignancy.

### Ethics Approval

The study protocol was approved by the hospital ethics committee (No. 202201002, Date: 2022-01-05).

Of 168 patients, 81 (control group) received continuous low flow oxygen inhalation. Briefly, patients received continuous low flow oxygen inhalation, and the oxygen flow was maintained at 2.5 L/minute. The remaining 87 patients (NIV group) received noninvasive ventilator assisted ventilation using the noninvasive ventilator (*Weinmann*, Germany). The positive end expiratory pressure combined with pressure support ventilation mode was adopted. The parameters were as follows:

- Respiratory ratio 1:1.5;
- The initial suction pressure of 8cm H_2_O that was gradually increased to 15cm H_2_O;
- The initial exhalation pressure of 2cm H_2_O that was gradually increased to 5cm H_2_O;
- The initial oxygen concentration of 80%, gradually reduced to 35%. The ventilation time and pressure were reasonably adjusted according to the patient’s condition. After the condition was stabilized, the patient was transitioned to catheter oxygen inhalation.

Medical records of all patients contained basic information and relevant indicators after treatment, such as: (1) blood gas analysis indicators of patients before and 48 hours after the treatment, including SaO_2_, PaO_2_, PaO_2_/FiO_2_ and PaCO_2_. (2) The serum indexes of patients before the treatment, and 24h and 48h after the treatment. Indexes were measured by collecting 3ml venous blood samples. The expression NT-pro BNP was done by fluorescence immunoassay, using mini-VIDAS fluorescence immunoanalyzer (BioMérieux, France). The expression of cTnI was done by electrochemiluminescence using Access electrochemiluminescence instrument (Beckman Coulter, USA).

### Statistical analysis

SPSS 22.0 was used for data processing, [n (%)] was used to represent non-grade counting data (the test method is *χ^2^*). (*X̅*±*S*) was used to represent measurement data (using t test). *P*<0.05 indicated statistically significant difference.

## RESULTS

A total of 168 patients met the inclusion criteria. Of them, 81 patients (45 males and 36 females) comprised the control group and 87 patients (45 males and 42 females) were included in the NIV group based on the mode of treatment received. The age of patients in the control group ranged from 35 to 79 years, with an average of (57.13±9.31) years. Classification of respiratory failure in the control group was as follows: 46 cases - Type-I and 35 cases - Type-II. In the NIV group, the age of patients ranged from 37 to 80 years, with an average of (58.63±10.44) years. Classification of respiratory failure in the NIV group was as follows: 45 cases - Type-I and 42 cases - Type-II. There was no significant difference in basic characteristics between the two groups (*P*>0.05), as shown in Table-I.

**Table I:** Comparison of general information between the two groups

Group	n	Gender (Male/Female)	Age (year)	Types of respiratory failure	

				Type-I	Type-II
Control group	81	45/36	57.13±9.31	46	35
NIV group	87	45/42	58.63±10.44	42	45
χ^2^ t	-	0.248	0.951	1.219	
P	-	0.619	0.343	0.270	

There was no significant difference in blood gas indexes between the two groups before treatment(*P*>0.05), After 48 hours of treatment, the blood gas analysis and treatment of the two groups have improved. After comparison between the groups, SaO_2_, PaO_2_ and PaO_2_/FiO_2_ were higher in the NIV group compared to the control group, while the expression of PaCO_2_ was lower than that in the control group (*P*<0.05) (Table-II).

**Table II:** Comparison of the blood gas analysis index of the two groups (*X̅*±s).

Group (n)	SaO_2_ (%)		PaO_2_ (mmHg)		PaO_2_/FiO_2_ (mmHg)		PaCO_2_ (mmHg)	
			
	Before therapy	After 48 hours of treatment	Before therapy	After 48 hours of treatment	Before therapy	After 48 hours of treatment	Before therapy	After 48 hours of treatment
Control group (n=81)	71.16±9.52	82.49±9.68^a^	64.19±7.86	76.61±7.97^a^	145.40±20.86	234.50±17.34^a^	69.70±4.73	55.16±6.04^a^
NIV group (n=87)	69.34±8.58	90.49±9.28^a^	62.44±8.42	82.21±7.98^a^	142.90±18.66	248.29±17.90^a^	68.52±5.47	44.37±6.24^a^
t	1.299	5.464	1.389	4.547	0.819	5.065	1.483	11.358
P	0.196	<0.001	0.167	<0.001	0.414	<0.001	0.140	<0.001

***Note:***

a compared with the same group before treatment *P*<0.05.

Before treatment, there was no significant difference in the expression of NT Pro BNP and cTnI between the two groups (*P*>0.05), but after 24 and 48 hours of treatment, the expression of NT-pro BNP and cTnI in both groups increased, but it was significantly lower in the NIV group patients compared to the control group (*P*<0.05) (Table-III).

**Table III:** Comparison of the serum index of the two groups (*X̅*±s).

Group (n)	NT-pro BNP expression (pg/ml)			cTnI expression (ng/ml)		
	
	Before therapy	After 24 hours treatment	After 48 hours treatment	Before therapy	After 24 hours treatment	After 48 hours treatment
Control group (n=81)	459.12±68.29	666.12±100.74^a^	573.27±94.34^a^	2.32±0.88	6.38±0.98^a^	5.84±1.00^a^
NIV group (n=87)	444.22±72.27	607.77±98.96^a^	509.19±102.42^a^	2.34±0.87	5.45±1.00^a^	4.55±1.03^a^
t	1.370	3.786	4.208	0.159	6.082	8.205
P	0.172	<0.001	<0.001	0.874	<0.001	<0.001

***Note:***

a compared with the same group before treatment *P*<0.05.

## DISCUSSION

Acute heart failure is characterized by its rapid onset and progression. After the onset, the patient’s ventricular end diastolic pressure level increases abnormally. At the same time, the cardiac output rapidly decreases, hindering the normal pulmonary vein return and leading to a significant increase in pulmonary vein pressure. As a result, a high volume of blood flows directly into the alveoli and pulmonary interstitium, resulting in pulmonary edema. The affected pulmonary ventilation will increase the risk of hypoxemia and can cause respiratory failure and increased oxygen consumption that may aggravate the degree of heart failure.^9^

Presently, the main focus of the acute heart failure treatment is to timely correct hypoxemia and improve pulmonary ventilation, ventilation function and the overall prognosis of patients.^10^ Continuous low flow oxygen inhalation is a common method of the clinical treatment of acute heart failure that is complicated by respiratory failure. It can alleviate the condition and inhibit the continuous progress of the disease to a certain extent, but it is less efficient in correcting hypoxemia. Noninvasive ventilator-assisted ventilation is gradually becoming more prevalent in clinical use in recent years. This ventilation technique uses nasal masks, and, as studies show, can effectively reduce respiratory muscle fatigue, improve ventilation, promote the reduction of respiratory work, and alleviate the degree of illness.^11^

In this study, noninvasive ventilator-assisted ventilation was applied to the treatment of patients with acute heart failure and respiratory failure. It was found that this treatment scheme can improve the curative effect of patients with acute heart failure and respiratory failure and promote the improvement of blood gas and serum indexes. Arterial oxygen saturation, SaO_2_, is an important indicator of respiratory circulation that can accurately evaluate the oxygen carrying capacity of hemoglobin and lung oxygenation. The partial pressure of oxygen, PaO_2,_ is the pressure produced by the physical dissolution of O_2_ molecules in plasma and can objectively reflect the status of pulmonary function. PaO_2_ / FiO_2,_ the ratio of PaO_2_ to the fraction of inspired oxygen, FiO_2,_ has been used to quantify the degree of abnormalities in pulmonary gas exchange. The partial pressure of carbon dioxide, PaCO_2_ is the pressure generated by dissolved CO2 molecules in plasma, and is an indicator of ventilation function of alveoli.^12^ Nakanishi M et al.^13^ found that abnormal values of SaO_2_, PaO_2_, PaO_2_/FiO_2_ and PaCO_2_ in patients with acute heart failure complicated with respiratory failure. In agreement with these results, and the results of Saillard C et al.,^14^ our study showed that SaO_2_, PaO_2_ and PaO_2_/FiO_2_ values in the NIV group after treatment were higher than those in the control group, while the value of PaCO_2_ was lower than that in the control group.

NT-pro BNP is a marker of heart failure. It has the long half-life and can be used as an objective indicator of cardiac function. NT-pro BNP expression is directly proportional to the degree of heart failure. CTnI is an important index to evaluate the therapeutic effect and prognosis of patients with heart disease.^15^ Therefore, in patients with acute heart failure complicated with respiratory failure, monitoring of NT-pro BNP and cTnI levels in addition to blood gas analysis indexes may help in inhibiting the progress and aggravation of heart failure. Our study showed that the expression of NT-pro BNP and cTnI in the NIV group was lower than that in the control group, which was consistent with the results of Westhoff M and De Palo M.^16^,^17^

Noninvasive ventilator-assisted ventilation technology is easy and safe. It allows to adjust the patient’s respiratory rate and breathing time according to the patient’s situation, inhibit airway resistance, improve the respiratory status and positive pressure ventilation level, promote the relief of respiratory muscle fatigue, prevent the collapse of bronchi, help to increase the level of SaO_2_ and accelerate the discharge rate of CO_2_. Noninvasive ventilator-assisted ventilation can also actively correct hypoxemia and improve blood gas analysis indexes, and control the level of thoracic negative pressure. The resulting improvement of coronary blood supply capacity and myocardial oxygen supply capacity will reduce the level of myocardial tension, reduce thoracic negative pressure, inhibit ventricular blood return, improve oxygen cooperation, and promote the overall improvement of cardiac function.

### Limitations

It includes small number of cases included, single nature of the disease, few observation indicators, and no long-term follow-up observation. Additionally, this is a single-center retrospective study, which makes it difficult to effectively control the interference factors.

## CONCLUSION

Noninvasive ventilator-assisted ventilation in patients with acute heart failure and respiratory failure leads to the significant improvement of blood gas and serum indexes. The use of this technique may improve the overall curative effect of ventilatory support.

### Authors’ Contributions:

**YW** conceived and designed the study.

**XH, NY, RZ and YS** collected the data and performed the analysis.

**YW** was involved in the writing of the manuscript and is responsible for the integrity of the study. All authors have read and approved the final manuscript.

## References

[1] Farooq F, Imran N, Abbas M (2021). Ivabradine effects on heart rate and quality of life among chronic heart failure patients. J Pak Med Assoc.

[2] Yu H, Chang J, Zhong W, Ye P (2018). Characteristics of clinical drugs for elderly chronic heart failure complicated with different degrees of renal insufficiency. Pak J Med Sci.

[3] Chebib N, Nesme P, Freymond N, Argaud L, Rimmelé T, Bohé J (2019). Acute Respiratory Failure in Obesity-Hypoventilation Syndrome Managed in the ICU. Respir Care.

[4] Nimdet K, Techakehakij W (2017). Congestive heart failure in children with pneumonia and respiratory failure. Pediatr Int.

[5] Haidl P, Jany B, Geiseler J, Andreas S, Arzt M, Dreher M (2020). Guideline for Long-Term Oxygen Therapy - S2k-Guideline Published by the German Respiratory Society. Pneumol Stuttg Ger.

[6] Van Ooteghem G, Dasnoy-Sumell D, Lambrecht M, Reychler G, Liistro G, Sterpin E (2019). Mechanically-assisted non-invasive ventilation:A step forward to modulate and to improve the reproducibility of breathing-related motion in radiation therapy. Radiother Oncol.

[7] vander Meer P, Gaggin HK, Dec GW (2019). ACC/AHA Versus ESC Guidelines on Heart Failure:JACC Guideline Comparison. J Am Coll Cardiol.

[8] Lentz S, Roginski MA, Montrief T, Ramzy M, Gottlieb M, Long B (2020). Initial emergency department mechanical ventilation strategies for COVID-19 hypoxemic respiratory failure and ARDS. Am J Emerg Med.

[9] Miller PE, Caraballo C, Ravindra NG, Mezzacappa C, McCullough M, Gruen J (2019). Clinical Implications of Respiratory Failure in Patients Receiving Durable Left Ventricular Assist Devices for End-Stage Heart Failure. Circ Heart Fail.

[10] Li H, Zhang F, Zhang D, Tian X (2021). Changes of Serum Ficolin-3 and C5b-9 in Patients with Heart Failure. Pak J Med Sci.

[11] Frat JP, Ricard JD, Quenot JP, Pichon N, Demoule A, Forel J (2019). Non-invasive ventilation versus high-flow nasal cannula oxygen therapy with apnoeic oxygenation for preoxygenation before intubation of patients with acute hypoxaemic respiratory failure:a randomised, multicentre, open-label trial. Lancet Respir Med.

[12] Park J, Lee YJ, Hong KS (2019). Proposed safe apnea test using positive end-expiratory pressure valve and short-term blood gas analysis:Observational study. Medicine (Baltimore).

[13] Nakanishi M, Miura H, Nakao K, Fujino M, Arakawa T, Fukui S (2019). Combination of Peak Exercise Systolic Blood Pressure and Left Atrial Diameter as a Novel Non-Spirometry Prognostic Predictor Comparable to Peak Oxygen Uptake for Heart Failure with Reduced Ejection Fraction. Circ.

[14] Saillard C, Mallet D, Chow-Chine L, Bisbal M, Faucher M, Sannini A (2020). Non-invasive ventilation indication for critically ill cancer patients admitted to the intensive care unit for acute respiratory failure (ARF) with associated cardiac dysfunction:Results from an observational study. PLOS One.

[15] Liu L, Liu S, Shen L, Tu B, Hu Z, Hu F (2020). Correlations between cardiac troponin I and nonsustained ventricular tachycardia in hypertrophic obstructive cardiomyopathy. Clin Cardiol.

[16] Westhoff M, Litterst P (2018). Ventilation Parameters under Adaptive Servo Ventilation:A Comparison on Behalf of CSA-Pattern, BNP/NT-pro-BNP, and Ejection Fraction. Respir Int Rev Thorac Dis.

[17] De Palo M, Guida P, Mastro F, Nanna D, Quagliara TAP, Rociola R (2017). Myocardial protection during minimally invasive cardiac surgery through right mini-thoracotomy. Perfusion.

